# Double-blind, randomized pilot clinical trial targeting alpha oscillations with transcranial alternating current stimulation (tACS) for the treatment of major depressive disorder (MDD)

**DOI:** 10.1038/s41398-019-0439-0

**Published:** 2019-03-05

**Authors:** Morgan L. Alexander, Sankaraleengam Alagapan, Courtney E. Lugo, Juliann M. Mellin, Caroline Lustenberger, David R. Rubinow, Flavio Fröhlich

**Affiliations:** 1 Department of Psychiatry, University of North Carolina at Chapel Hill, Chapel Hill, NC 27599 USA; 2 Carolina Center for Neurostimulation, University of North Carolina at Chapel Hill, Chapel Hill, NC 27599 USA; 3 Neural Control of Movement Lab, Department of Health Sciences and Technology, ETH Zurich, Zurich, 8092 Switzerland; 4 Department of Neurology, University of North Carolina at Chapel Hill, Chapel Hill, NC 27599 USA; 5 Department of Biomedical Engineering, University of North Carolina at Chapel Hill, Chapel Hill, NC 27599 USA; 6 Department of Cell Biology and Physiology, University of North Carolina at Chapel Hill, Chapel Hill, NC 27599 USA; 7 Neuroscience Center, University of North Carolina at Chapel Hill, Chapel Hill, NC 27599 USA

## Abstract

Major depressive disorder (MDD) is one of the most common psychiatric disorders, but pharmacological treatments are ineffective in a substantial fraction of patients and are accompanied by unwanted side effects. Here we evaluated the feasibility and efficacy of transcranial alternating current stimulation (tACS) at 10 Hz, which we hypothesized would improve clinical symptoms by renormalizing alpha oscillations in the left dorsolateral prefrontal cortex (dlPFC). To this end, 32 participants with MDD were randomized to 1 of 3 arms and received daily 40 min sessions of either 10 Hz-tACS, 40 Hz-tACS, or active sham stimulation for 5 consecutive days. Symptom improvement was assessed using the Montgomery–Åsberg Depression Rating Scale (MADRS) as the primary outcome. High-density electroencephalograms (hdEEGs) were recorded to measure changes in alpha oscillations as the secondary outcome. For the primary outcome, we did not observe a significant interaction between treatment condition (10 Hz-tACS, 40 Hz-tACS, sham) and session (baseline to 4 weeks after completion of treatment); however, exploratory analyses show that 2 weeks after completion of the intervention, the 10 Hz-tACS group had more responders (MADRS and HDRS) compared with 40 Hz-tACS and sham groups (*n* = 30, *p* *=* 0.026). Concurrently, we found a significant reduction in alpha power over the left frontal regions in EEG after completion of the intervention for the group that received per-protocol 10 Hz-tACS (*n* = 26, *p* < 0.05). Our data suggest that targeting oscillations with tACS has potential as a therapeutic intervention for treatment of MDD.

## Introduction

Major depressive disorder (MDD) is a common, severe psychiatric illness that has a lifetime prevalence of about 16.6% in adults^1^ and results in the highest burden of disability among all mental and behavioral disorders^2^. Current recommended drug therapies are associated with suboptimal remission rates and, oftentimes, undesirable side effects^3^. Furthermore, the effects of pharmacological agents are widespread; in contrast, interventions that can target specific abnormalities in brain activity may permit greater therapeutic precision. Patients with MDD exhibit elevated oscillatory activity, specifically in the alpha frequency band (8–12 Hz)^4^, which is often localized to left frontal regions, resulting in what has been called “frontal alpha asymmetry”^5^. Although alpha oscillations serve important functions in the healthy brain^6,7^, increased alpha oscillation strength in depressed patients represents a state of neuronal hypoactivity leading to disrupted affective processing^8^. Thus, renormalizing this elevated alpha activity could potentially mitigate symptoms of MDD. Brain activity can be altered with noninvasive brain stimulation methods such as transcranial magnetic stimulation (TMS) and transcranial electric stimulation. Rhythmic TMS bursts at the alpha frequency can entrain brain activity in healthy humans^9^, and synchronized TMS at individualized alpha frequencies has been evaluated for the treatment of MDD^10^, although TMS has not yet been demonstrated to alter alpha oscillations in these patients.

Another stimulation paradigm used to modulate endogenous brain activity is transcranial alternating current stimulation (tACS), which applies a weak electric current with a sine-wave pattern to the scalp. Such sine-wave stimulation may better lend itself to targeting oscillatory brain activity than methods such as TMS. TACS can modulate cortical oscillations that mediate cognitive function^11^ and can selectively modulate oscillations at the applied frequency^12,13^. In addition, the side effects of tACS are mild and transient, and no serious adverse events have yet been reported^14^. Despite these promising results, tACS has not yet been tested as a possible therapeutic intervention for MDD. To address this gap in knowledge, we hypothesized that tACS at 10 Hz targeting both left and right frontal areas with synchronous stimulation would improve the symptoms of MDD and restore a more physiological balance of alpha oscillations by reducing the pathologically elevated power of the alpha oscillation in left dorsolateral prefrontal cortex (dlPFC).

To test this hypothesis, we conducted a pilot double-blind study to evaluate the feasibility, safety, and efficacy of tACS as a treatment for the symptoms of depression. Patients diagnosed with MDD were randomized to one of three arms to compare 10 Hz-tACS, 40 Hz-tACS, and active sham stimulation. The investigation of a second, different stimulation frequency (i.e., 40 Hz) served to assess whether symptom and electrophysiological changes were frequency-dependent or merely stimulation-dependent. The tACS intervention comprised 40 min of daily stimulation for 5 consecutive days. The primary outcome was the change in Montgomery–Åsberg Depression Rating Scale (MADRS) score from baseline to the final follow-up study visit 4 weeks after completion of the intervention. To understand how tACS affects brain activity, we measured alpha power changes using high-density electroencephalography as our secondary outcome.

## Materials and methods

This study was a double-blind, randomized, sham-controlled pilot clinical trial conducted at The University of North Carolina at Chapel Hill from May 2015 to June 2017 and registered at ClinicalTrials.gov (NCT02339285). The study was approved by the Biomedical Institutional Review Board at UNC Chapel Hill (IRB # 14-1622) and used a Data Safety Monitoring Board (DSMB) through the North Carolina Translational & Clinical Studies Institute, to ensure participant safety. Bi-annual reviews of blinded data and adverse events were submitted to the DSMB. All participants provided written informed consent before all study-related activities.

### Participants

A total of 32 patients (27 female; aged 36.69 ± 13.08 years) diagnosed with unipolar, non-psychotic MDD (confirmed with the M.I.N.I. International Neuropsychiatric Interview 7.0 for the Diagnostic Statistical Manual of Mental Disorders, 5th Edition), with a Hamilton Depression Rating Scale (HDRS) of >8 and low suicide risk, defined as scoring <3 on the Suicide Item on the HDRS, were randomized in this trial. Previous treatments include medication (94% reported) and therapy (69% reported), indicating the enrolled participants in this sample have attempted to treat their depression before enrollment. Of the 32 enrolled participants (defined as intent-to-treat, or ITT, sample), 26 completed all study visits as designed (defined as per-protocol, or PP, sample; see CONSORT). Screened participants were excluded from participation for the following reasons: concurrent anticonvulsant medications or daily treatments with benzodiazepines (limited as-needed use that was discontinued more than 48 h before a study session was allowed); diagnosis of alcohol or substance dependence (other than nicotine) within the last 12 months; current Axis I mood or psychotic disorder other than MDD; lifetime comorbid psychiatric bipolar or psychotic disorder; eating disorder (current or within the past 6 months); obsessive-compulsive disorder (lifetime); post-traumatic stress disorder (current or within the last 6 months); attention-deficit/hyperactivity disorder (currently under treatment); history of significant head injury or traumatic brain injury, prior brain surgery, or any brain devices/implants; history of seizures, unstable medical illness, or pregnancy. Although not an exclusion criterion, none of the participants were left-handed (Edinburgh Handedness Inventory, 82.8 ± 24.9). Screened participants were not excluded for use of antidepressants and 38% of participants were on at least one antidepressant at the time of enrollment. To control for changes in medication, participants were required to be at least 6 weeks stable on their antidepressants. See Table S1 for further baseline demographics on all randomized participants.

### Study schedule

Participants who completed the study attended a total of eight sessions (Fig. S1). Inclusion and exclusion criteria were assessed with a preliminary phone screening and then more extensively at the initial session with the study coordinator. At the initial session, participants signed consent and completed several questionnaires (demographics, Edinburgh handedness Inventory, “Hunter Beliefs About Treatment Questionnaire,” used with permission of the UCLA Laboratory of Brain, Behavior, and Pharmacology, ©2005, 2017 UC Regents). In addition, the study coordinator administered the M.I.N.I. and the HDRS to confirm eligibility. Before randomization, eligible participants also met with an experienced mood disorders clinician (D.R.R.) to further assess their clinical symptoms and to verify the participants met the inclusion criteria. Once eligibility was confirmed, participants returned for 5 consecutive days of treatment (Day 1 to Day 5). Baseline scores for all assessments were completed on Day 1. Participants also attended a 2-week follow-up and a 4-week follow-up after they completed the week of stimulation.

### Randomization

Participants were randomized into three study arms (10 Hz-tACS, *n* = 10; 40 Hz-tACS, *n* = 11; and active sham at 10 Hz, *n* = 11). Intervention type was based on study codes prepared by a member of the research lab, who was not otherwise associated with the study, and codes were randomized such that no more than three participants in a row received the same intervention. All authors and members of the research team were unaware of the group assignments until completion of the entire study. To administer stimulation in a double-blind manner, we developed a custom Matlab-controlled computer interface (Mathworks, Natick, MA; NIDaq USB 6001, National Instruments, TX, USA) to control two Neuroconn DC plus stimulators (Neuroconn Ltd., Ilmenau, Germany) that delivered the stimulation based on the study code entered. To ensure that the correct waveform was applied for each session, this interface recorded the applied waveform for subsequent verification by a group member not associated with the study.

### Stimulation

**Fig. 1:**
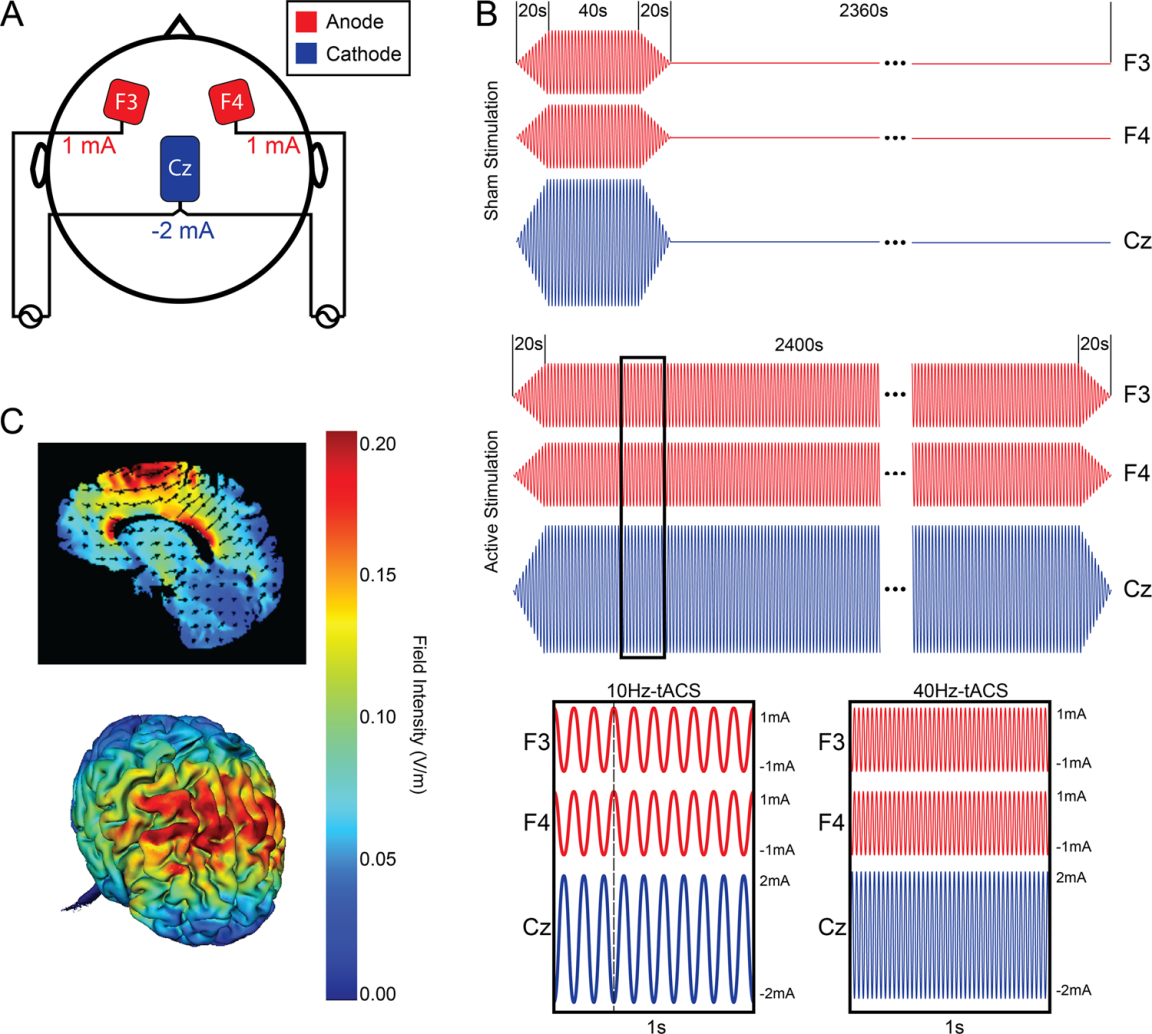
**a** Stimulation configuration for all participants. Two stimulators were used; one connected to the electrode over F3, one connected to the electrode over F4, and both connected to the electrode over Cz. The red electrodes (F3 and F4) are the anode and the blue return electrode over Cz is the cathode. **b** Sham and active stimulation paradigms. Ramp-in and ramp-out is 20 s for all conditions, with 40 s of active stimulation for Sham stimulation, 2400 s of active stimulation for 10 Hz-tACS and 40 Hz-tACS. Anodes (F3 and F4) and cathode (Cz) are at opposite phase at any given point during stimulation. **c** Electric field simulation: 2D (top) and 3D (bottom) representation (HD-Explore, Soterix Medical, New York, NY, USA)

All three study arms used the same electrode montage (Fig. 1). Three electrodes with Ten20 paste (Bio-Medical Instruments, Clinton Township, Michigan) were applied to the scalp. Two 5 × 5 cm electrodes were placed over the left and right frontal areas (F3 and F4, respectively, in the 10–20 placement system) with a third 5 × 7 cm “return/reference” electrode placed over the vertex (Cz in the 10–20 system). The electrode montage described here delivers in-phase synchronized stimulation to both the left and right frontal regions to target the imbalance between frontal alpha activity.

Each participant completed 5 consecutive days of the intervention (40 min of stimulation) at approximately the same time of day (±90 min). The choice of intervention duration (5 consecutive days, 40 min each day) was informed by a previous study of transcranial direct current stimulation, which found efficacy 4 weeks after completion of treatment^15^. The tACS stimulation waveform was a sine-wave with an amplitude of 2 mA at Cz and an amplitude of 1 mA at F3 and F4 (amplitudes are reported as zero-to-peak). There were two tACS conditions: the proposed therapeutic frequency of 10 Hz and the control frequency of 40 Hz. Previous research indicates that gamma oscillations have a stronger relationship to cognition^16^, and would theoretically not target alpha oscillations and not result in mood symptom changes; therefore, 40 Hz-tACS would be an appropriate control frequency for this trial. Active sham stimulation included 20 s of ramp-in to 40 s of 10 Hz-tACS, with a ramp-out of 20 s, for a total of 80 s of stimulation. Both 10 Hz and 40 Hz-tACS included 20 s of ramp-in to 40 min of stimulation, with a ramp-out of 20 s for a total of 2440 s of stimulation (Fig. 1b). During each stimulation session, participants were seated comfortably upright with their eyes open and were asked to focus on a ReefScapes video (Undersea Productions, Queensland, Australia) presented on a large projector screen directly in front of them. This video served the purpose of masking the phosphenes induced by tACS and keeping all participants in the same state during stimulation sessions. On the final day of stimulation (Day 5), participants were asked whether they believed they received stimulation over the past week (Yes, No, I don’t know) to assess blinding.

### High-density electroencephalography

Resting-state EEG (RSEEG) was collected at Day 1 (baseline), Day 5, and the 4-week follow-up, using a 128 channel EEG system (Geodesic EEG system 410, Electrical Geodesics, Inc., OR, USA). RSEEG was administered before the final stimulation on Day 5 to avoid recording the immediate aftereffects of tACS. Participants followed pre-programmed computer-generated instructions (Presentation, Neurobehavioral Systems, CA, USA) and had their eyes closed for 2 min, following which they had their eyes open for 2 min^17^. This sequence was repeated twice resulting in a total of 8 min of RSEEG. The sequence was counterbalanced across participants, i.e., half of the participants started with 2 min of eyes-open condition and the other half started with 2 min of eyes-closed condition. During the eyes-open condition, participants were instructed to fixate on a cross-hair. Participants also completed a working memory task, the results of which are not presented here.

EEG analysis was performed using EEGLab^18^ and custom-written Matlab scripts. Preprocessing consisted of band-pass filtering to 1–50 Hz, downsampling to 250 Hz, removal of bad channels based on low correlations to surrounding channels, and artifact subspace reconstruction^19^ followed by independent component analysis^20^ to remove artifacts caused by eye blinks, eye movements, muscle activity, and heartbeats. EEG data were separated according to the eyes-open and eyes-closed condition, and epoched into 10 s segments, following which power spectral density was estimated using multi-taper windowed fast Fourier transform method^21^. To determine changes in RSEEG at Day 5 and the 4-week follow-up, spectral power in the alpha frequency band (8–12 Hz) was calculated and decibel-normalized to spectral power estimated from baseline at each individual electrode as well as averages within a topographical region. The former was used to assess significant changes in oscillation power within each group, whereas the latter was used to compare changes across the three groups. In addition, alpha power in baseline session was log-transformed and compared between the groups.

### Side effects and safety

Side effects were assessed after every stimulation session. Suicidal thoughts/actions were monitored daily with a self-report questionnaire, starting from baseline until the 4-week follow-up as well as during clinical assessments (MADRS and HDRS). Possible development of mania was monitored with the Young Mania Rating Scale (YMRS) administered at every study visit from baseline until the 4-week follow-up. The Montreal Cognitive Assessment (MoCA) was administered at two time points (baseline, 4-week follow-up) to assess any possible cognitive changes.

### Outcome measures

The primary outcome measure was defined as the change in depressive symptoms measured by the MADRS from baseline to the 4-week follow-up for the ITT sample. The MADRS was administered before stimulation at baseline, after the fifth stimulation on Day 5, at the 2-week follow-up, and the 4-week follow-up. The secondary outcome was the change in raw alpha power measured at the 4-week follow-up relative to baseline for the PP sample. The choice of the 4-week follow-up as the primary outcome was based on a previous trial using electrical stimulation^15^. Exploratory outcome measures were defined as the change in the HDRS and Beck Depression Inventory (BDI). Response to treatment was defined as at least a 50% reduction in symptoms from baseline for each clinical assessment^22^. Remission for the MADRS was defined as scoring ≤9, remission for the HDRS was defined as scoring ≤7, and remission for the BDI was defined as scoring ≤12^23^.

### Statistical analysis

Custom-written scripts in R (R Foundation for Statistical Computing, Vienna, Austria) were used for analysis and are available by request. Libraries used in R included lme4^24^ and pbkrtest^25^. Differences in demographics and baseline characteristics of the three study arms and the severity of adverse effects were assessed with one-way analysis of variance (ANOVA), *χ*^2^-tests of independence, and pairwise *t*-test with false discovery rate (FDR) correction. Spearman’s rank-order correlation was used to assess the possible role of placebo response in the effects observed using a belief of treatments questionnaire, as the data was non-parametric. To assess equality of variance, we used Bartlett’s K-squared. We used a linear mixed model analysis with fixed factors of “session” (baseline, the 4-week follow-up) and “condition” (10 Hz-tACS, 40 Hz-tACS, active sham 10 Hz-tACS), with random factor “participant” to account for repeated measures within participants. The choice of a linear mixed model for the main outcome takes into consideration any missing data in our ITT sample. The interaction between “session” and “condition” is defined as the effects of “session” on “condition”. Kenward–Roger approximations were used to calculate *p*-values and perform F-tests for each factor and their interaction in the mixed model. Effect sizes between groups were calculated using eta-squared (*η*^2^), and effect sizes within groups (baseline to Day 5, 2-week follow-up, 4-week follow-up) were calculated using Cohen’s *d*. Differences in rates of response and remission of the three study arms and the success of blinding to treatment arm were assessed with *χ*^2^-tests of independence.

Statistical analysis of EEG data was performed using custom R scripts and the “lmertest” package^26^, which allows fitting linear mixed-effects model and uses Satterthwaite’s approximation to degrees of freedom, to determine the F statistics of the fixed effects. Alpha power changes were averaged across electrodes over different topographical regions (frontal, central, occipital, temporal/parietal; Fig. S2A). Alpha asymmetry was measured as $\ln \left( {\frac{{right\,alpha\,power}}{{left\,alpha\,power}}} \right)$ of the pooled average of the left and right frontal electrodes. Linear mixed-effects models were fit with power change as the dependent variable, topographical region and condition as fixed factors and participant as random factor. The residuals of the models were tested for normality using the Shapiro–Wilk test. Two different models were fit for Day 5 and the 4-week follow-up. Post-hoc analysis was performed to get contrasts adjusted using Tukey’s honest significant difference (HSD) in the “emmeans” package. Baseline alpha power differences were determined by fitting a linear model with log-transformed baseline alpha power as a dependent variable, and condition and region as factors. To determine which electrodes exhibited significant power change, we performed a one-sample *t*-test with FDR correction. Spearman’s correlation coefficient was computed between log-transformed baseline alpha scores and changes.

### Sample size determination

The target sample size was 30 participants, with *n* = 10 for each arm of the study. This sample was chosen based on funding duration and a focus on feasibility, as this was the first study to use tACS in this population; however, several studies of tACS in healthy populations used a similar sample size to show changes in alpha^27,28^. A total of 32 participants were randomized (ITT sample), with 26 completing all study sessions (PP sample). Enrollment ended because funding had ended.

### Code availability

All codes used to analyze the presented results are available upon request.

## Results

### Primary outcome (MADRS)

In the ITT analysis, MADRS scores for all three groups decreased significantly from baseline to the 4-week follow-up, but there was no significant difference in these changes based upon condition (10 Hz-tACS, 40 Hz-tACS, active sham 10 Hz-tACS) (i.e., there was a significant effect of session (F_1,28.618_ = 38.87, *p* < 0.001), but not condition (F_2,28.681_ = 0.22, *p* = 0.80) or interaction (F_2,26.559_ = 0.65, *p* = 0.53)).

### Secondary outcome (EEG)

**Fig. 2:**
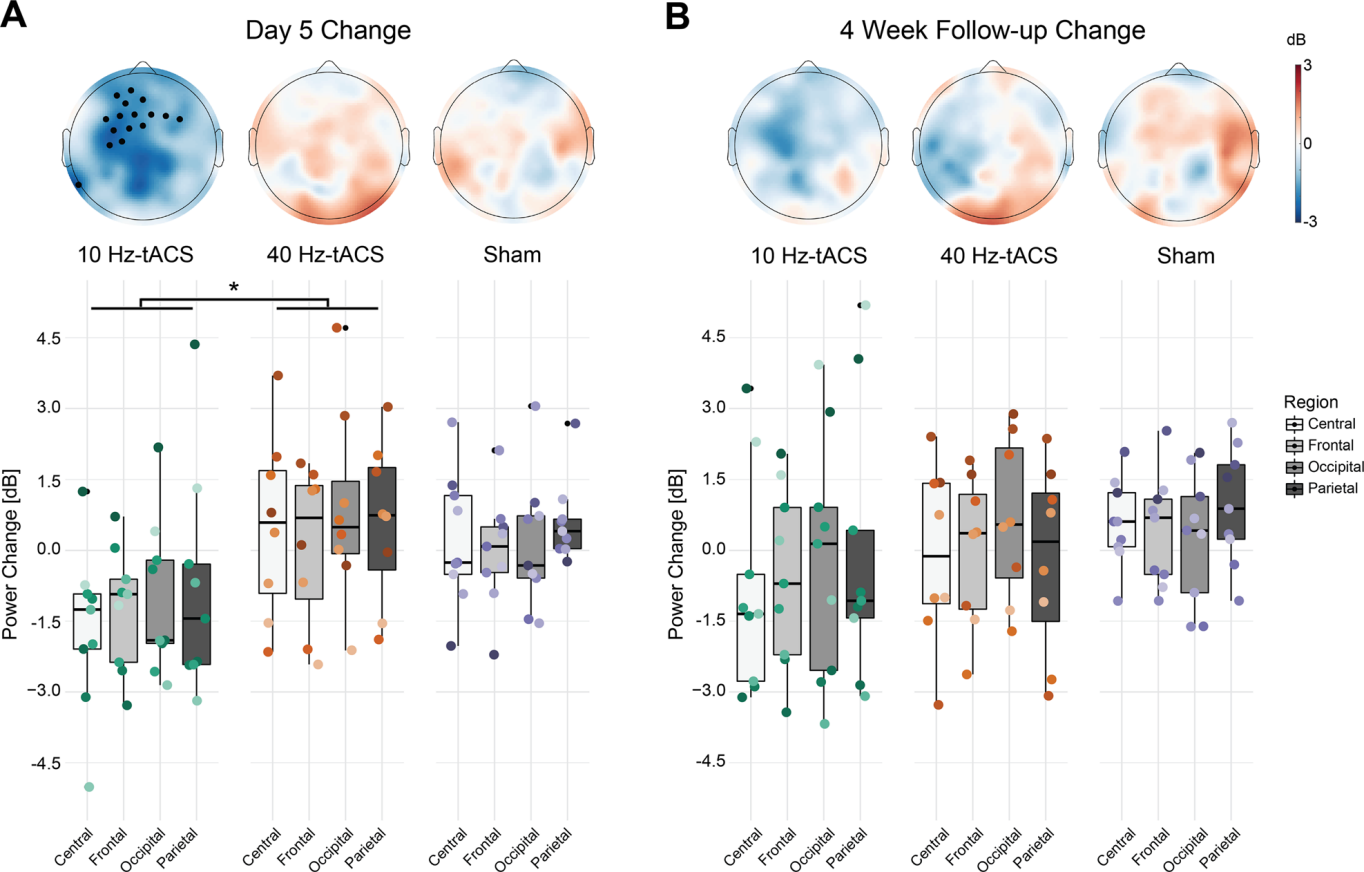
Changes in EEG alpha power in eyes-open condition. **a** Topographical distribution of power change at Day 5 and F2. Black filled circles denote electrodes that showed significant change relative to Day 1. **b** Mean alpha power change at topographical region level at Day 5 and F2. *Statistical significance at *α* = 0.05

To verify whether tACS was effective in engaging alpha oscillations, we assessed changes in resting-state alpha power at Day 5 (Fig. 2a) and the 4-week follow-up (Fig. 2b) in the PP sample. We performed statistical analysis of alpha power change at individual electrode level and at region level. Baseline alpha power was not different between the three groups (Fig. S2C; linear model factor condition F_2,92_ = 0.126, *p* = 0.881; factor region F_3,92_ = 3.727, *p* = 0.014, interaction F_6,92_ = 0.042, *p* = 0.99) nor did we find significant alpha asymmetry in our participant sample (0.0199 ± 0.0336 (mean ± SEM), *p* = 0.5584 one-sample *t*-test) or within any treatment groups (10 Hz-tACS: −0.0040 ± 0.0592, *p* = 0.9474; 40 Hz-tACS: 0.0585 ± 0.0548, *p* = 0.3211; sham: 0.0096 ± 0.0639, *p* = 0.8847). Post-hoc analysis of contrasts in factor region revealed that the difference between frontal and occipital regions (*p* = 0.020) and the difference between occipital and parietal regions were significant (*p* *=* 0.039), whereas the difference between central and occipital regions were trend-level (*p* = 0.087). At individual electrode level, the 10 Hz-tACS group showed significant decrease over the left frontal regions (black circles in Fig. 2a, *p* < 0.05, one-sample *t*-test with FDR correction), whereas no effect of stimulation was observed in the other groups at Day 5 or in any of the groups at the 4-week follow-up. We did not find any significant differences between the three stimulation conditions on frontal alpha asymmetry on Day 5 relative to Day 1 (F_2,23_ = 0.24, *p* = 0.7852 one-way ANOVA) nor did we find any significant effect of stimulation on Day 5 relative to Day 1 within any treatment groups (10 Hz-tACS: 0.0144 ± 0.0330, *p* = 0.6738; 40 Hz-tACS: 0.0145 ± 0.0246, *p* = 0.5739; sham: −0.0171 ± 0.0477, *p* = 0.7289). We observed significant negative correlations between the log-transformed baseline alpha power and alpha power changes at Day 5 in all the regions (Fig. S2B; Spearman’s rank correlations, Frontal *ρ* = −0.45, *p* = 0.02; Central *ρ* = −0.54, *p* < 0.01; Parietal *ρ* = −0.65, *p* < 0.01; Occipital *ρ* = −0.55, *p* < 0.01). Thus, we included the log-transformed baseline alpha power as a covariate in our analysis of the effects of the intervention. To assess the effect of stimulation on alpha power on Day 5, we fitted a linear mixed model with power change in alpha band as dependent variable and condition (3 levels—10 Hz-tACS, 40 Hz-tACS, and Sham) and regions (4 levels—frontal, central, parietal, and occipital) as fixed factors and participants as random factors. ANOVA of the model revealed significant effect of condition (F_2,21.595_ = 3.931, *p* = 0.035) and region (F_3,79.358_ = 7.762, *p* < 0.001). There was no significant effect of interaction (F_6,68.284_ = 1.073, *p* = 0.388). Post-hoc analysis of contrasts in factor condition using Tukey’s HSD revealed significant difference between 10 Hz-tACS group and 40 Hz-tACS group (*p* = 0.039). For factor region, significant differences were found between frontal and occipital (*p* < 0.001), central and occipital (*p* < 0.01), and frontal and parietal (*p* < 0.05), whereas the difference between occipital and parietal was trend-level (*p* < 0.1). ANOVA of the linear mixed model for alpha power changes at the 4-week follow-up revealed significant effect of only region (F_3,80.094_ = 2.921, *p* = 0.039). There was no significant effect of condition (F_2,22.722_ = 0.629, *p* = 0.542), whereas interaction was trend-level (F_6,69.170_ = 2.019, *p* = 0.075). Post-hoc analysis revealed that the differences between frontal and occipital, and central and occipital were trend-level (*p* < 0.1). At individual electrode level, the 10 Hz-tACS group showed significant decrease from baseline to Day 5 over the left frontal regions (black circles in Fig. 2a, *p* < 0.05, one-sample *t*-test with FDR correction), whereas no effect of stimulation was observed in the other groups at Day 5 or in any of the groups at the 4-week follow-up. There was no statistically significant correlation between change in alpha asymmetry and change in MADRS (*ρ* = 0.0316, *p* = 0.8784) nor between change in frontal alpha oscillations and change in MADRS (*ρ* = 0.1865; *p* = 0.6397). Taken together, our results indicate that 10 Hz-tACS was effective in targeting alpha oscillations predominantly in the frontal and central regions, but this change did not have a relationship with change in clinical symptoms. Similar analysis of eyes-closed data did not reveal any significant effect of stimulation in alpha power change.

### Response and remission (MADRS, HDRS, BDI)

**Fig. 3:**
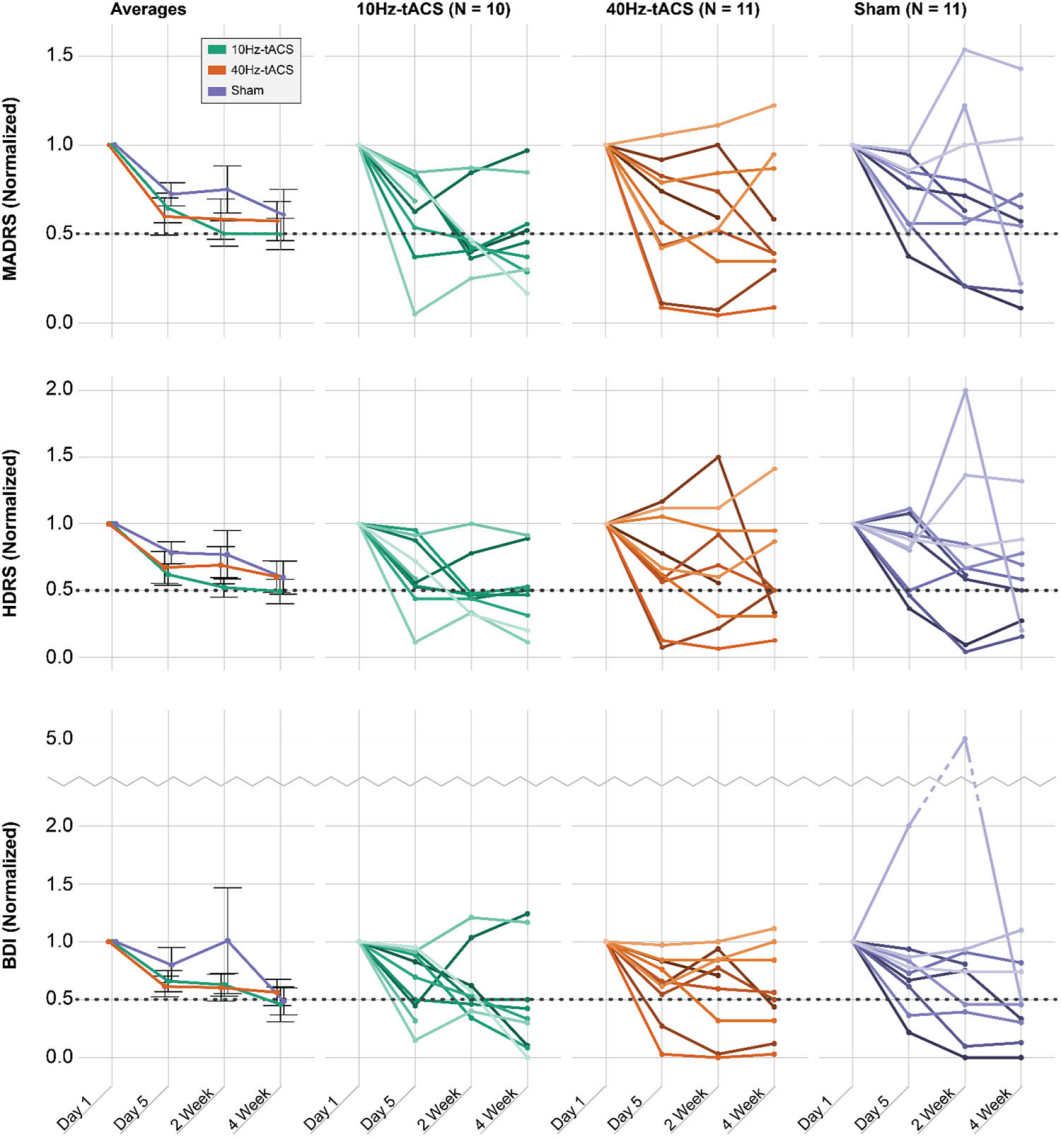
Individual scores per participant for the MADRS, HDRS, and BDI. Scores are normalized based on a ratio in comparison to baseline scores. Averages include SE bars. Dashed line in each figure represents the threshold for response (i.e., at least a 50% reduction in symptoms from baseline). Note that in the nine graphs on the right, each line represents an individual participant. In the 10 Hz-tACS group, one person withdrew from participation after Day 5; in the 40 Hz-tACS group, one person was lost to follow-up during the stimulation week and one person withdrew from participation after the 2-week follow-up; in the sham group, one person withdrew from participation during the stimulation week and one person withdrew from participation after the 2-week follow-up

Within the ITT sample, we found a significant relationship between condition and response rates for the MADRS (*χ*^2^ = 7.334, df = 2, *p* = 0.026) and HDRS (*χ*^2^ = 7.334, df = 2, *p* = 0.026) at the 2-week follow-up. This indicates that the 10 Hz-tACS group had a higher rate of response at the 2-week follow-up (77.8%) than the 40 Hz-tACS (30.0%) and sham stimulation groups (20.0%) (Fig. 3). No other significant relationships were found for response rates (Table 1). We also looked into remission rates over the course of the intervention; however, around half of participants did not qualify for remission at any time point as measured by the MADRS (59% did not remit), the HDRS (50% did not remit), and the BDI (41% did not remit). No significant relationships were found for remission rates (Table 1).

**Table 1:** Response (the number of participants that achieved at least a 50% reduction in symptoms compared with baseline) and remission rates at each time for each assessment and each time point

	MADRS, *n* (%)		HDRS, *n* (%)		BDI, *n* (%)	
	Response	Remission	Response	Remission	Response	Remission
**Day 5**						
10 Hz-tACS (*n* = 10)	2 (20.0)	1 (10.0)	2 (20.0)	2 (20.0)	4 (40.0)	2 (20.0)
40 Hz-tACS (*n* = 10)	4 (40.0)	3 (30.0)	2 (20.0)	4 (40.0)	2 (20.0)	5 (50.0)
Sham (*n* = 10)	2 (20.0)	2 (20.0)	3 (30.0)	3 (30.0)	2 (20.0)	3 (30.0)
*χ* ^*2*^	1.364	1.25	0.373	0.953	1.364	2.100
*df*	2	2	2	2	2	2
*p*	0.506	0.535	0.83	0.621	0.506	0.350
**2-Week follow-up**						
10 Hz-tACS (*n* = 9)	7 (77.8)	1 (11.1)	7 (77.8)	4 (44.4)	4 (44.4)	5 (55.6)
40 Hz-tACS (*n* = 10)	3 (30.0)	3 (30.0)	3 (30.0)	3 (30.0)	3 (30.0)	4 (40.0)
Sham (*n* = 10)	2 (20.0)	2 (20.0)	2 (20.0)	4 (40.0)	4 (40.0)	4 (40.0)
*χ* ^*2*^	7.334	1.034	7.334	0.448	0.448	0.607
*df*	2	2	2	2	2	2
*p*	0.026*	0.596	0.026*	0.800	0.800	0.738
**4-Week follow-up**						
10 Hz-tACS (*n* = 9)	5 (55.6)	3 (33.3)	5 (55.5)	4 (44.4)	7 (77.8)	7 (77.8)
40 Hz-tACS (*n* = 10)	5 (50.0)	6 (60.0)	6 (60.0)	5 (50.0)	5 (50.0)	5 (50.0)
Sham (*n* = 9)	3 (33.3)	3 (33.3)	4 (44.4)	6 (66.7)	6 (66.7)	6 (66.7)
*χ* ^*2*^	0.973	1.867	0.482	0.973	1.625	1.625
*df*	2	2	2	2	2	2
*p*	0.615	0.393	0.786	0.615	0.444	0.444

Note that the MADRS and HDRS assess symptoms from the past week, whereas BDI assesses symptoms from the past 2 weeks. Each clinical assessment has different requirements for remission: remission for the MADRS is 9 or less, remission for the HDRS is 7 or less, and remission for the BDI is 12 or less. + denotes *p* < 0.10 and * denotes *p* < 0.05

### Effect sizes (MADRS, HDRS, BDI)

In exploratory analyses, we calculated effect sizes between and within the treatment groups in the ITT sample. We looked at the mean (SD) change in MADRS from baseline to Day 5, the 2-week follow-up, and the 4-week follow-up (Table S2). The change in scores from baseline to the 4-week follow-up demonstrated a moderate effect size between conditions (*η*^2^ = 0.060) and the largest effect size was seen in the change from baseline to the 2-week follow-up (*η*^2^ = 0.116). We also calculated Cohen’s *d* in each treatment group from baseline to Day 5, the 2-week follow-up, and the 4-week follow-up. For the MADRS, the largest within-group effect size was seen in the 10 Hz-tACS group from baseline to the 2-week follow-up (*d* = 1.70), with the next largest seen in the 10 Hz-tACS group from baseline to the 4-week follow-up (*d* = 1.60).

We next sought to validate the effect of tACS on improving MDD symptoms by including analogous rating scales. The HDRS was used as a complementary outcome measure to the MADRS and the scores were strongly correlated (*R*^2^ = 0.85, *p* < 0.001). The mean (SD) change in HDRS from baseline to the 4-week follow-up was −8.67 (5.32) for 10 Hz-tACS, −5.20 (5.67) for 40 Hz-tACS, and −5.11 (7.61) for sham stimulation. Similar to the MADRS, this change demonstrated a moderate between-group effect size (*η*^2^ = 0.071). For the HDRS, the largest within-group effect size was seen in the 10 Hz-tACS group from baseline to the 4-week follow-up (*d* = 1.61), with the next largest seen in the 10 Hz-tACS group from baseline to the 2-week follow-up (*d* = 1.58).

In addition, the BDI was collected as a self-report measure of symptom changes and was strongly correlated with the MADRS (*R*^2^ = 0.72, *p* < 0.001). The mean (SD) change in BDI from baseline to the 4-week follow-up was −14.78 (13.14) for 10 Hz-tACS, −12.20 (11.68) for 40 Hz-tACS, and −12.33 (10.71) for sham stimulation. This change demonstrated a small between-group effect size (*η*^2^ = 0.011). For the BDI, the largest within-group effect size was seen in the 10 Hz-tACS group at the 4-week follow-up (*d* = 1.54), with the next largest seen in the sham group from baseline to the 2-week follow-up (*d* = 1.44).

### Safety

We used one-way ANOVAs to determine group differences in the experience and expectations of side effects (Table S3). Participants from all three groups reported minimal side effects and there was no difference between the three groups with the exception of “flickering lights” (or phosphenes, *p* = 0.014). We ran post-hoc paired *t*-tests with FDR correction and found that there was not a significant difference in this side effect between 10 Hz-tACS and 40 Hz-tACS (*p* = 0.99); however, there was a trend-level difference between 10 Hz-tACS and sham (*p* = 0.09), and a significant difference between 40 Hz-tACS and sham (*p* < 0.01).

After enrollment, four participants in the sham stimulation group experienced an increase in suicidal ideation and one of these four participants reported suicidal plans (i.e., scoring >3 on the suicide item on the HDRS). No participants in the 10 Hz-tACS and 40 Hz-tACS groups experienced an increase in suicidal ideation from baseline during the course of the study.

No participants developed mania or hypomania at any point during study participation based on the YMRS. Two adverse events were reported during the course of the study; however, after a thorough investigation, neither were determined to be related to the study or intervention. Finally, participants from all three groups experienced a small improvement in cognition from baseline to the 4-week follow-up as measured by the MoCA (Table S2). There was no significant difference in this improvement in relation to condition across sessions (F_2,25.717_ = 1.354, *p* = 0.276).

### Blinding

Self-report responses from participants on whether they thought they received verum stimulation (Yes, No, I don’t know) and the treatment group (10 Hz-tACS, 40 Hz-tACS, sham) were significantly related (*χ*^2^ = 8.304, df = 2, *p* = 0.016). Those who received 40 Hz-tACS were more likely to think they had been stimulated, with 90% correctly reporting that they received stimulation. For those who received 10 Hz-tACS, 40% reported that they received stimulation; for those who received sham stimulation, 30% reported that they had received stimulation.

### Expectations of treatment

We ran one-way ANOVAs on expectations of treatment (Item 1: expected likelihood of symptom improvement; Item 2: expected improvement in symptoms) to assess whether there were group differences in these expectations of treatment, and there were not (*p* = 0.409, *p* = 0.990, respectively). There was no significant relationship between either of these items and the percent change in MADRS score from baseline to the 2-week follow-up (Item 1: *ρ* = 0.343, *p* = 0.054; Item 2: *ρ* = 0.317, *p* = 0.078) and from baseline to the 4-week follow-up (Item 1: *ρ* = 0.273, *p* = 0.131; Item 2: *ρ* = 0.243, *p* = 0.181). Post-hoc analysis shows that the trend-level relationship between expectations and percent change in MADRS from baseline to the 2-week follow-up was driven by the sham group (Item 1: *ρ* = 0.778, *p* = 0.005; Item 2: *ρ* = 0.693, *p* = 0.018), but not the 10 Hz-tACS (Item 1: *ρ* = −0.072, *p* = 0.843; Item 2: *ρ* = 0.114, *p* = 0.753) or 40 Hz-tACS groups (Item 1: *ρ* = 0.363, *p* = 0.272; Item 2: *ρ* = 0.080, *p* = 0.816).

## Discussion

This pilot clinical trial was designed to evaluate the feasibility, safety, and preliminary efficacy of tACS as a treatment for the symptoms of MDD. Although our primary outcome measure, change in MADRS at the 4-week follow-up in the ITT sample, was not significantly different between the groups, 10 Hz-tACS significantly outperformed 40 Hz-tACS and sham stimulation in terms of response rates at the 2-week follow-up. These results were consistent in both clinician-administered measures (MADRS, HDRS). In addition, 10 Hz-tACS was also effective in engaging alpha oscillations compared with sham or 40 Hz-tACS. Taken together, our results suggest that targeting alpha oscillations with tACS is a potentially viable therapeutic approach and a fully powered subsequent study is justified to further establish tACS as a treatment for depression.

Stimulation was tolerated well by the participants, with no serious adverse events related to the stimulation reported and no development of mania, hypomania, or increase in suicidal ideation as a result of stimulation. Retention rates were high for all three groups. These data indicate that future trials using tACS are feasible in this population.

Our physiological target in this study was frontal alpha oscillations. Altered alpha oscillations are thought to be a key physiological marker in patients with MDD. Resting state recordings in patients with MDD are characterized by increased synchrony in the theta and alpha frequency bands^4,29^, the latter of which likely emerges from abnormal thalamocortical connectivity^30^. In addition, alpha activity in the left frontal regions has been shown to be higher than that of the right frontal regions^5,31,32^. This increased, asymmetric alpha activity is thought to reflect reduced neuronal activity in the left dlPFC, one of several key regions identified as abnormal in brain imaging studies of depression^8,33^. Although repetitive TMS (rTMS) studies for treating depression often use stimulation frequencies around the alpha band^34^, the effect of rTMS on oscillations in patients with MDD has seldom been demonstrated, except in a few case studies^35,36^. The 10 Hz-tACS led to significantly reduced alpha oscillations over the left frontal regions along with the highest response rates, suggesting that successful reshaping of disrupted oscillations may have led to the decreased symptoms observed, despite the fact that alpha asymmetry was not observed in our sample. This indicates that 10 Hz-tACS may have a therapeutic effect regardless of asymmetry. However, our interpretation of the results is limited by the fact that we did not collect RSEEG data at the 2-week follow-up. In studies with healthy volunteers, tACS has often been suggested to increase alpha power immediately after stimulation with effects lasting up to 70 min^27,28,37^. In contrast, our results indicate a decrease in alpha power. The differences could be attributed to dosage (five sessions of 40 min stimulation vs. one session of 20 min stimulation). Alternatively, the differences could be due to the altered network oscillations in patients with MDD (i.e., the impact of tACS may differ in the presence of abnormal alpha activity). The exact mechanism of tACS has not yet been determined. Studies suggest tACS could induce entrainment of cortical oscillations or plasticity (or likely a combination of both)^12,28,38^. The effects of periodic perturbation have been shown to be state-dependent, further confounding the possible mechanism^27,39,40^. Although the immediate after-effect of tACS may be enhancement in alpha power, repeated application of tACS may lead to a resetting of oscillators potentially through homeostatic mechanisms resulting in a decrease in alpha power, as is proposed for rTMS^41^. Further studies are required to determine the mechanisms underlying the effect of tACS in patients with aberrant network oscillations.

Despite the promising results as measured by clinician-administered assessments (MADRS, HDRS), we did not see the same results in the self-report measure of the BDI. This may be due to the different time frame (past week vs. past 2 weeks) or that patients may have a delay in recognizing symptom changes compared to clinicians.

This study has several limitations. First, this was an exploratory pilot study with a small sample size, powered to detect only large effect sizes. Although we determined our sample sizes based on previous tACS results, our study investigated effects at a longer time frame compared with the previous studies. Second, blinding was not successful in the 40 Hz-tACS group; however, the blinding was successful in the 10 Hz-tACS group, indicating that, at least in this group, the change in symptoms may be more related to the stimulation and less likely a placebo effect. Third, further studies with EEG data collection before and after each stimulation session will be required to strengthen the evidence for alpha oscillations as the target for the 10 Hz-tACS intervention. Finally, as this was a small pilot study, the heterogeneity introduced by medication, therapy, duration of illness, etc., may have affected our results.

To our knowledge, this is the first time tACS has been studied for the treatment of MDD, demonstrating that it may be a feasible and efficacious treatment in this population. With these results, the next steps would be to validate these results in a larger sample that can establish the use of tACS to treat MDD. Future directions may also include investigating dosage, maintenance treatment, and following participants for longer than 4 weeks after treatment.

## Supplementary information

Fig. S1

Fig. S2

Table S1

Table S2

Table S3

CONSORT
