# Supplementary material for: Double-blind, randomized pilot clinical trial targeting alpha oscillations with transcranial alternating current stimulation (tACS) for the treatment of major depressive disorder (MDD)
Source: Transl Psychiatry. 2019 Mar 5;9:106. doi: 10.1038/s41398-019-0439-0 (PMC6401041; doi:10.1038/s41398-019-0439-0)
Supplement: Supplementary file 1 — Fig. S1 [file 41398_2019_439_MOESM1_ESM.docx]

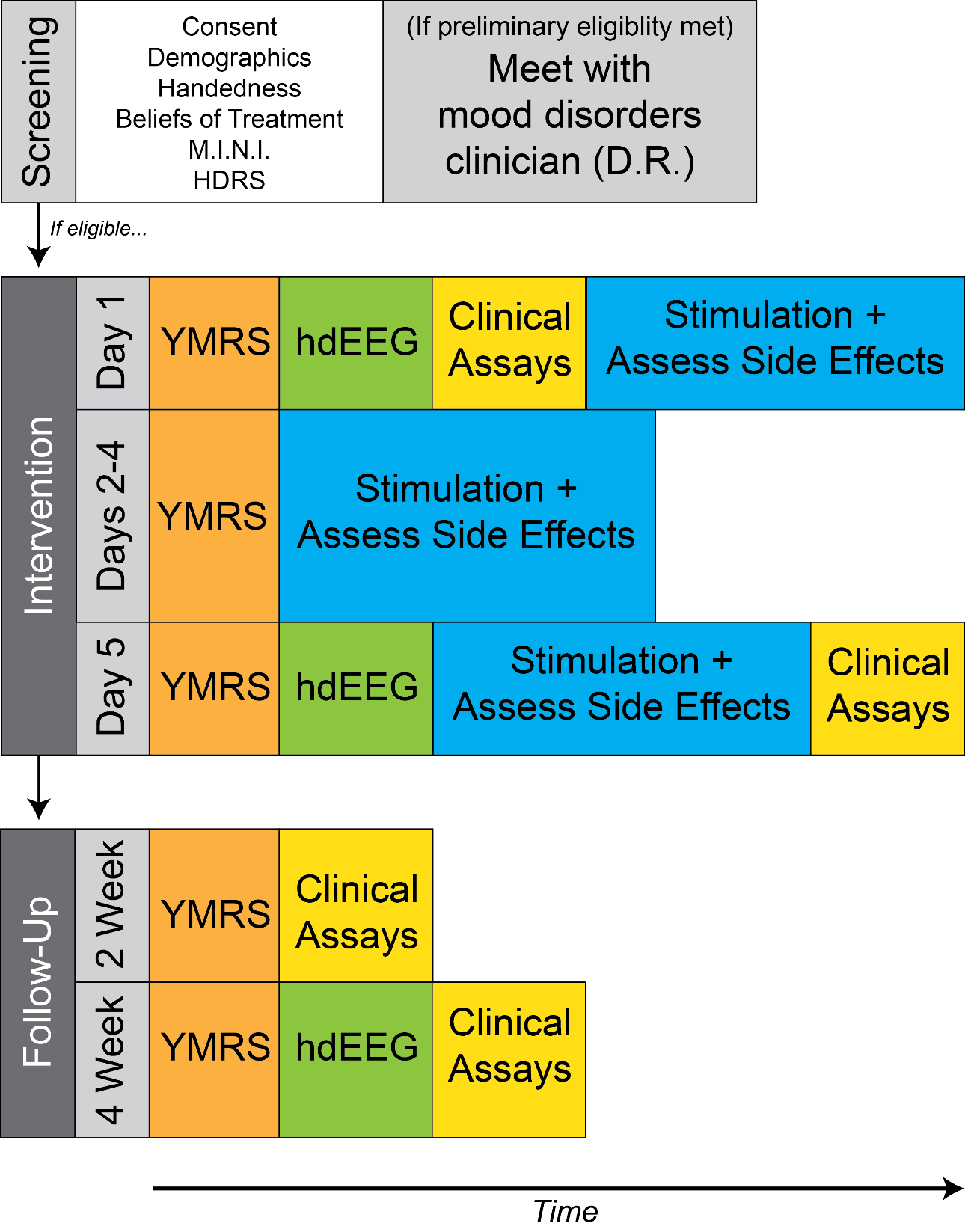


**Figure S1.** Diagram of the study schedule. The Young Mania Rating Scale (YMRS) was administered at the start of every session. Note that stimulation on Days 2-4 started 1 hour after that on Days 1 and 5 to ensure that all stimulation sessions were within 90 minutes of each other. Clinical assays were administered within 20 minutes after the final stimulation on Day 5.
