# Supplementary material for: Double-blind, randomized pilot clinical trial targeting alpha oscillations with transcranial alternating current stimulation (tACS) for the treatment of major depressive disorder (MDD)
Source: Transl Psychiatry. 2019 Mar 5;9:106. doi: 10.1038/s41398-019-0439-0 (PMC6401041; doi:10.1038/s41398-019-0439-0)
Supplement: Supplementary file 2 — Fig. S2 [file 41398_2019_439_MOESM2_ESM.docx]

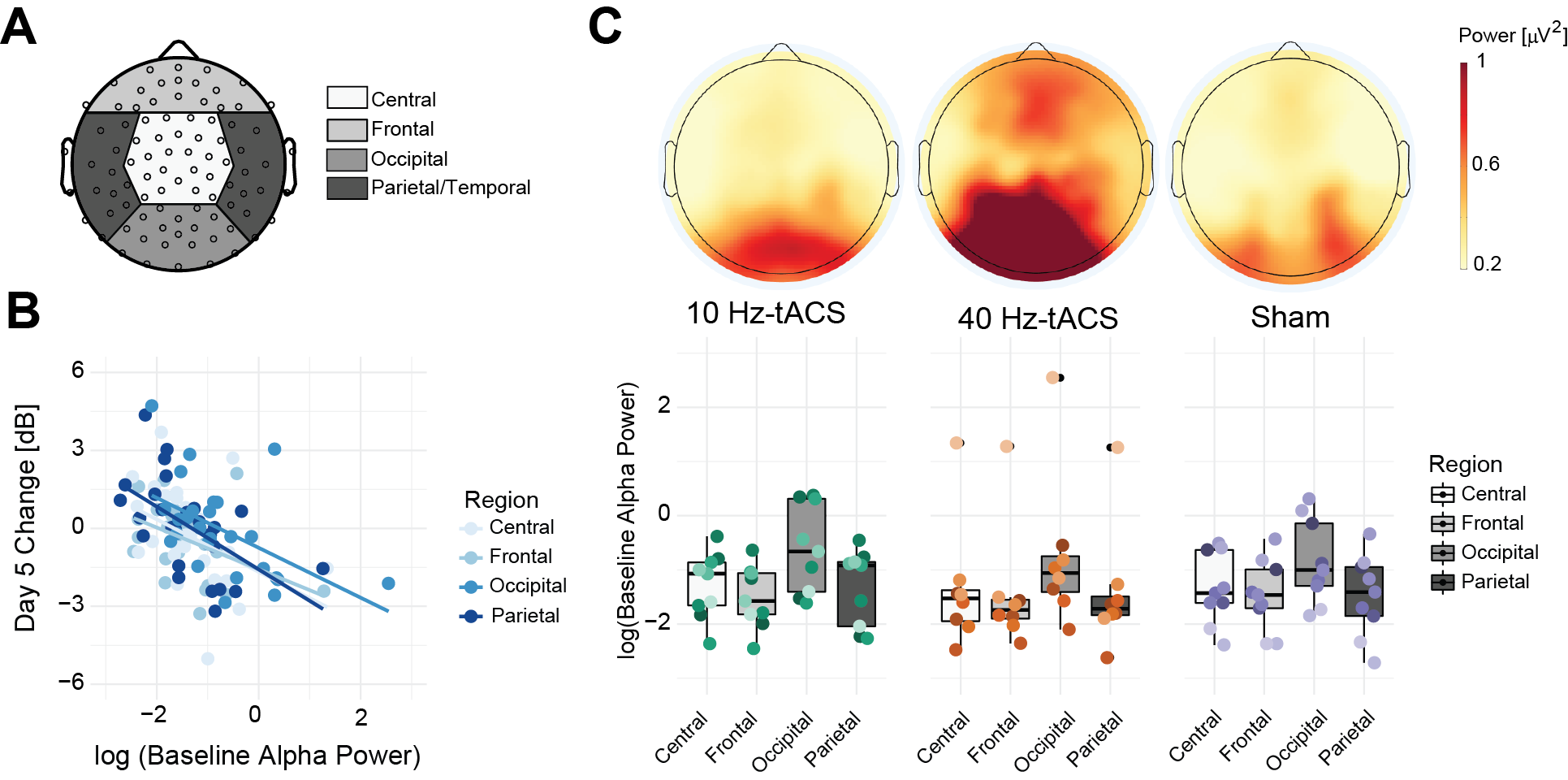


**Figure S2**. (A) Topographical regions used in analysis of EEG data and location of EEG electrodes. (B) Change in alpha power at Day 5 plotted against baseline alpha power along with lines of best fit for each topographical region. Each dot denotes a participant. (C) Mean baseline alpha power measured across the scalp and at different topographical regions
