# Supplementary material for: Double-blind, randomized pilot clinical trial targeting alpha oscillations with transcranial alternating current stimulation (tACS) for the treatment of major depressive disorder (MDD)
Source: Transl Psychiatry. 2019 Mar 5;9:106. doi: 10.1038/s41398-019-0439-0 (PMC6401041; doi:10.1038/s41398-019-0439-0)
Supplement: Supplementary file 3 — Table S1 [file 41398_2019_439_MOESM3_ESM.docx]

|  | 10Hz-tACS (N=10) | 40Hz-tACS (N=11) | Sham (N=11) | *Statistical Test* | *df* |
| --- | --- | --- | --- | --- | --- |
| Age, mean (SD) | 36.3 (15.2) | 35.4 (11.6) | 38.4 (13.5) | *p* = 0.868 |  |
| Female, No. (%) | 9 (90.0) | 9 (81.8) | 9 (81.8) | χ^2^ = 0.349  *p* = 0.840 | 2 |
| Education Level, No. (%) |  |  |  |  |  |
| *High School Diploma (or Equivalent)* | 4 (40.0) | 0 (0.0) | 2 (18.2) |  |  |
| *Undergraduate Degree* | 2 (20.0) | 4 (36.4) | 4 (36.4) |  |  |
| *Advanced Degree* | 4 (40.0) | 5 (45.5) | 5 (45.5) | χ^2^ = 8.927 |  |
| *Other* | 0 (0.0) | 2 (18.2) | 0 (0.0) | *p* = 0.178 | 6 |
| Symptom Onset, No. (%) |  |  |  |  |  |
| *1-2 years ago* | 1 (10.0) | 1 (9.1) | 3 (27.3) |  |  |
| *3-5 years ago* | 0 (0.0) | 1 (9.1) | 1 (9.1) |  |  |
| *5-10 years ago* | 5 (50.0) | 4 (36.4) | 3 (27.3) |  |  |
| *10-15 years ago* | 0 (0) | 2 (18.2) | 0 (0.0) | χ^2^ = 7.179 |  |
| *>15 years ago* | 4 (40.0) | 3 (27.3) | 4 (36.4) | *p* = 0.518 | 8 |
| Antidepressant Use*, No. (%) |  |  |  |  |  |
| *SSRI* | 2 (20.0) | 1 (9.1) | 4 (36.4) |  |  |
| *SNRI* | 1 (10.0) | 0 (0.0) | 1 (9.1) |  |  |
| *Tetracyclic* | 1 (10.0) | 0 (0.0) | 0 (0.0) |  |  |
| *SARI* | 0 (0.0) | 1 (9.1) | 0 (0.0) |  |  |
| *Aminoketone* | 2 (20.0) | 1 (9.1) | 2 (18.2) |  |  |
| *Lithium* | 1 (10.0) | 0 (0.0) | 0 (0.0) |  |  |
| *2+ Antidepressants* | 1 (10.0) | 1 (9.1) | 2 (18.2) |  |  |
| *None* | 3 (30.0) | 8 (72.7) | 6 (54.5) |  |  |
| *Not reported* | 2 (20.0) | 1 (9.1) | 0 (0.0) |  |  |
| Benzodiazepine Use (as needed), No. (%)** | 1 (11.1) | 0 (0.0) | 0 (0.0) |  |  |
| Previous Treatments, No. (%) |  |  |  |  |  |
| Medication | 10 (100.0) | 9 (81.8) | 11 (100.0) |  |  |
| Therapy (e.g., CBT) | 8 (80.0) | 6 (54.5) | 8 (72.7) |  |  |
| Lifestyle Changes (e.g., exercise, diet) | 1 (10.0) | 5 (45.5) | 6 (54.5) |  |  |
| Alternative Therapies (e.g., acupuncture, meditation, mindfulness, prayer, light therapy) | 1 (10.0) | 2 (18.2) | 3 (27.3) |  |  |
| Other (e.g., ECT, neurofeedback) | 0 (0.0) | 3 (27.3) | 0 (0.0) |  |  |
| Alcohol Use |  |  |  |  |  |
| *Response ‘Yes’, No. (%)* | 8 (80.0) | 4 (36.4) | 8 (72.7) | χ^2^ = 5.004 *p* = 0.082 | 2 |
| *If ‘Yes’, # of drinks per week, mean (SD)* | 4.4 (2.8) | 7.5 (8.5) | 2.8 (2.0) |  |  |
| Expectations of Treatment*** |  |  |  |  |  |
| Expected likelihood of symptom improvement | 4.0 (1.2) | 4.8 (1.5) | 4.3 (1.5) | 0.409 |  |
| Expected improvement in symptoms | 4.0 (1.6) | 4.1 (1.5) | 4.0 (1.9) | 0.990 |  |

**Table S1.** Demographics. All demographics were assessed at the initial session. *Antidepressant use during the trial was received through self-report. The number of participants on antidepressants in comparison to those not on antidepressants was not different between the three treatment arms (χ^2^ = 3.431, df = 2, *p* = 0.180). **Participants were excluded for daily use of benzodiazepines; however, participants were permitted benzodiazepines on an as-needed basis. ***Expectations of Treatment were measured on a 9-point likert scale (Expected likelihood of symptom improvement: 1=not at all likely, 5=somewhat likely, 9=very likely; Expected reduction in symptoms: 1=no improvement, 5=moderate improvement, 9=marked improvement). One-way ANOVAs were performed on “Age” and “Expectations of Treatment” and χ^2^ tests of independence were performed on all other demographic information. No significant differences were found between all 3 treatment groups.
