# Supplementary material for: Double-blind, randomized pilot clinical trial targeting alpha oscillations with transcranial alternating current stimulation (tACS) for the treatment of major depressive disorder (MDD)
Source: Transl Psychiatry. 2019 Mar 5;9:106. doi: 10.1038/s41398-019-0439-0 (PMC6401041; doi:10.1038/s41398-019-0439-0)
Supplement: Supplementary file 4 — Table S2 [file 41398_2019_439_MOESM4_ESM.docx]

|  |  | **10Hz-tACS** | | | | **40Hz-tACS** | | | | **Sham** | | | |  |
| --- | --- | --- | --- | --- | --- | --- | --- | --- | --- | --- | --- | --- | --- | --- |
|  |  | *n* | Mean | (SD) | *d* | *n* | Mean | (SD) | *d* | *n* | Mean | (SD) | *d* | *η ^2^* |
| ***MADRS*** | *Baseline* | 10 | 28.80 | (6.36) |  | 11 | 25.00 | (7.21) |  | 11 | 24.55 | (5.18) |  | 0.089 |
|  | *Day 5 Δ* | 10 | -9.40 | (5.42) | 1.14 | 10 | -9.70 | (8.17) | 1.01 | 10 | -6.80 | (5.35) | 1.37 | 0.043 |
| *2 Week Follow-up Δ* | | 9 | -14.22 | (5.67) | 1.70 | 10 | -10.10 | (8.97) | 1.00 | 10 | -6.40 | (11.66) | 0.82 | 0.116 |
| *4 Week Follow-up Δ* | | 9 | -14.44 | (7.72) | 1.60 | 10 | -9.80 | (8.93) | 0.92 | 9 | -9.33 | (11.75) | 1.04 | 0.060 |
| ***HDRS*** | *Baseline* | 10 | 17.90 | (4.51) |  | 11 | 14.64 | (3.53) |  | 11 | 14.55 | (6.12) |  | 0.099 |
|  | *Day 5 Δ* | 10 | -6.00 | (3.09) | 1.09 | 10 | -4.90 | (5.49) | 0.97 | 10 | -3.40 | (4.60) | 0.72 | 0.059 |
| *2 Week Follow-up Δ* | | 9 | -8.22 | (4.74) | 1.58 | 10 | -5.10 | (5.84) | 1.07 | 10 | -4.40 | (8.87) | 0.69 | 0.061 |
| *4 Week Follow-up Δ* | | 9 | -8.67 | (5.32) | 1.61 | 10 | -5.20 | (5.67) | 0.95 | 9 | -5.11 | (7.61) | 0.76 | 0.071 |
| ***BDI*** | *Baseline* | 10 | 26.60 | (5.58) |  | 11 | 28.27 | (8.81) |  | 11 | 26.18 | (9.74) |  | 0.013 |
|  | *Day 5 Δ* | 10 | -8.60 | (6.42) | 1.17 | 10 | -10.60 | (10.07) | 1.20 | 10 | -7.90 | (7.16) | 1.04 | 0.022 |
| *2 Week Follow-up Δ* | | 9 | -10.22 | (8.58) | 1.43 | 10 | -11.70 | (12.40) | 1.25 | 10 | -9.90 | (11.03) | 1.21 | 0.006 |
| *4 Week Follow-up Δ* | | 9 | -14.78 | (13.14) | 1.54 | 10 | -12.20 | (11.68) | 1.26 | 9 | -12.33 | (10.71) | 1.44 | 0.011 |
| ***MoCA*** | *Baseline* | 10 | 28.20 | (1.99) |  | 11 | 27.27 | (1.62) |  | 11 | 27.82 | (2.48) |  | 0.036 |
| *4 Week Follow-up Δ* | | 9 | +0.56 | (0.53) | -0.18 | 10 | +1.40 | (1.35) | -0.84 | 9 | +1.33 | (1.58) | -0.58 | 0.095 |

**Table S2.** Scores from the MADRS, HDRS, BDI, and MoCA for the ITT sample summarized in a table; the first row for each assessment is the baseline score and the following rows are the change from baseline (negative scores indicate a reduction from baseline). We found no significant differences in the variances for each group in any assessment (MADRS: K^2^ = 1.91, df = 2, *p* > 0.1; HDRS: K^2^ = 0.96, df = 2, *p* > 0.1; BDI: K^2^ = 1.19, df = 2, *p* > 0.1; MoCA: K^2^ = 1.62, df = 2, *p* > 0.1). Note that higher scores in the MADRS, HDRS, and BDI indicate more severe depressive symptoms. The MoCA is scored out of a total possible 30 points, higher scores indicate better cognition, and scores of 26 or higher are considered normal. Within group effect size was calculated using paired Cohen’s *d* (baseline compared to Day 5, 2 Week Follow-up, 4 Week Follow-Up, with pooled standard deviation) and between group effect size was calculated using eta-squared (*η^2^*).
