# Supplementary material for: Double-blind, randomized pilot clinical trial targeting alpha oscillations with transcranial alternating current stimulation (tACS) for the treatment of major depressive disorder (MDD)
Source: Transl Psychiatry. 2019 Mar 5;9:106. doi: 10.1038/s41398-019-0439-0 (PMC6401041; doi:10.1038/s41398-019-0439-0)
Supplement: Supplementary file 5 — Table S3 [file 41398_2019_439_MOESM5_ESM.docx]

|  | **10Hz-tACS** | | **40Hz-tACS** | | **Sham** | |  |
| --- | --- | --- | --- | --- | --- | --- | --- |
| **S3.1 Side Effect** | Mean | (SD) | Mean | (SD) | Mean | (SD) | *p* |
| Headache | 1.22 | (0.34) | 1.27 | (0.39) | 1.32 | (0.47) | 0.852 |
| Neck pain | 1.08 | (0.19) | 1.27 | (0.60) | 1.18 | (0.33) | 0.577 |
| Scalp pain | 1.52 | (0.61) | 1.40 | (0.59) | 1.32 | (0.61) | 0.764 |
| Tingling | 2.06 | (0.65) | 1.89 | (0.48) | 1.84 | (0.48) | 0.618 |
| Itching | 1.90 | (0.63) | 1.45 | (0.51) | 1.45 | (0.57) | 0.140 |
| Ringing/Buzzing noise | 1.12 | (0.32) | 1.02 | (0.06) | 1.00 | (0.00) | 0.277 |
| Burning sensation | 1.74 | (0.41) | 1.69 | (0.56) | 1.67 | (0.66) | 0.960 |
| Local redness | 1.06 | (0.19) | 1.09 | (0.24) | 1.02 | (0.06) | 0.644 |
| Sleepiness | 2.22 | (0.68) | 2.22 | (0.64) | 2.24 | (0.71) | 0.998 |
| Trouble concentrating | 1.34 | (0.42) | 1.36 | (0.54) | 1.65 | (0.69) | 0.371 |
| Improved mood | 1.38 | (0.32) | 1.36 | (0.40) | 1.18 | (0.24) | 0.310 |
| Worsening of mood | 1.00 | (0.00) | 1.00 | (0.00) | 1.02 | (0.06) | 0.398 |
| Dizziness | 1.02 | (0.06) | 1.02 | (0.06) | 1.00 | (0.00) | 0.595 |
| Flickering Lights | 1.74 | (0.86) | 1.63 | (0.46) | 1.04 | (0.12) | 0.014* |
| **S3.2 Expectations of Side Effects** |  |  |  |  |  |  |  |
| Expected likelihood of developing side effects | 2.7 | (1.3) | 2.0 | (1.3) | 3.1 | (1.7) | 0.220 |
| Expected severity of side effects | 2.7 | (1.3) | 1.5 | (0.7) | 2.0 | (1.4) | 0.063^+^ |

**Table S3.1** Side effects as reported from all participants. Responses are on a 4-point likert scale (1=absent, 2=mild, 3=moderate, 4=severe). **S3.2** Expectations of Side Effects were measured on a 9-point likert scale (Expected likelihood of developing side effects: 1=not at all likely, 5=somewhat likely, 9=very likely; Expected severity of side effects: 1=minimal side effects, 5=moderate side effects, 9=severe side effects). “+” denotes p < 0.10 and “*” denotes p < 0.05.
