# Supplementary material for: Double-blind, randomized pilot clinical trial targeting alpha oscillations with transcranial alternating current stimulation (tACS) for the treatment of major depressive disorder (MDD)
Source: Transl Psychiatry. 2019 Mar 5;9:106. doi: 10.1038/s41398-019-0439-0 (PMC6401041; doi:10.1038/s41398-019-0439-0)
Supplement: Supplementary file 6 — CONSORT [file 41398_2019_439_MOESM6_ESM.pdf]

CONSORT

TRANSPARENT REPORTING of TRIALS

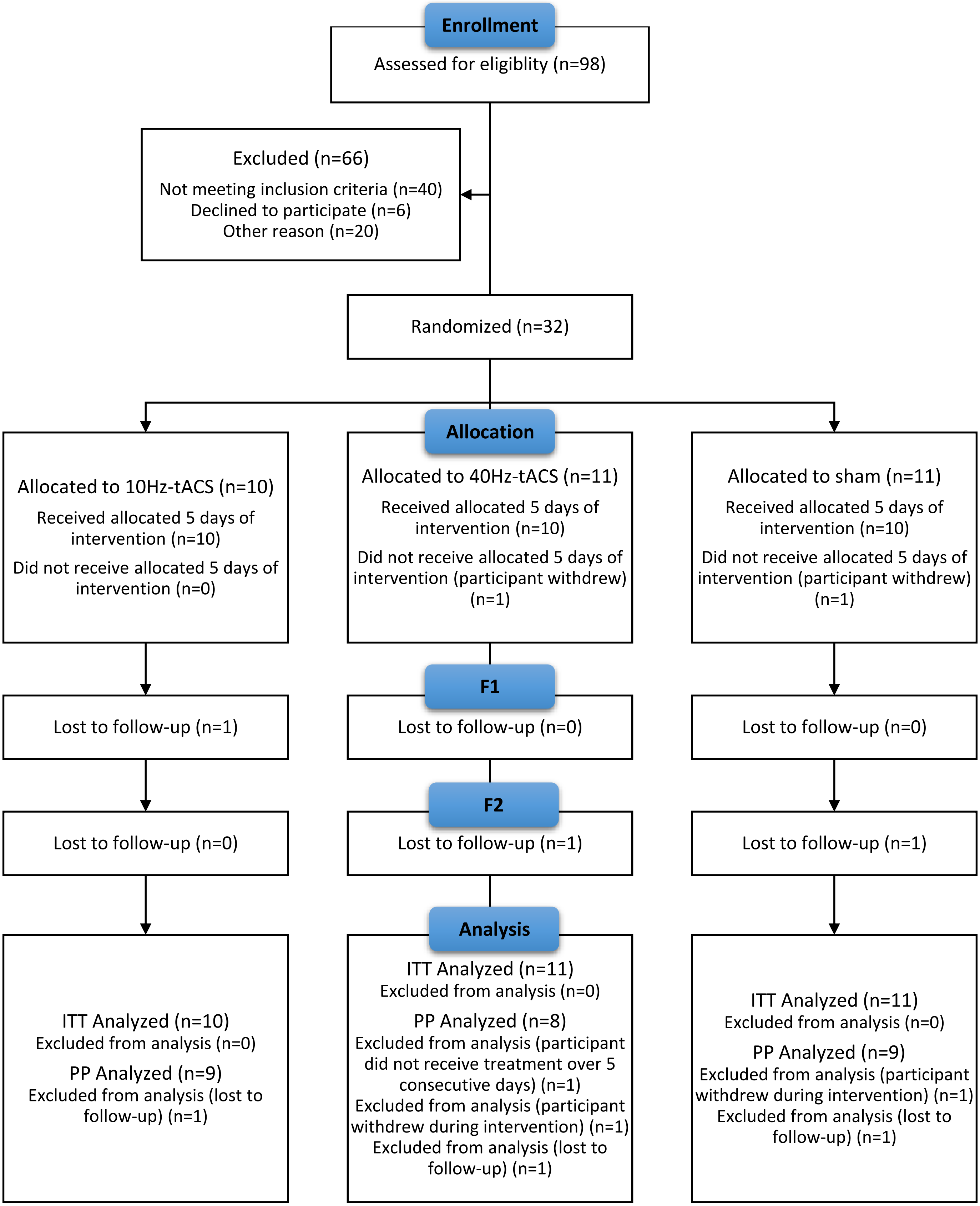

ITT = intent-to-treat
PP = per protocol

F1 = follow-up 1, 2 weeks after intervention
F2 = follow-up 2, 4 weeks after intervention
